# Relationship of Pre-season Training Load With In-Season Biochemical Markers, Injuries and Performance in Professional Soccer Players

**DOI:** 10.3389/fphys.2019.00409

**Published:** 2019-04-12

**Authors:** Sullivan Coppalle, Guillaume Rave, Abderraouf Ben Abderrahman, Ajmol Ali, Iyed Salhi, Sghaier Zouita, Amira Zouita, Matt Brughelli, Urs Granacher, Hassane Zouhal

**Affiliations:** ^1^M2S (Movement, Sport and Health Sciences Laboratory), University of Rennes 2, Rennes, France; ^2^Stade Lavallois Mayenne Football Club, Laval, France; ^3^Department of Performance, Université d’Angers, Angers, France; ^4^ISSEP Ksar Said, University of Manouba, Manouba, Tunisia; ^5^School of Sport, Exercise and Nutrition, Massey University, Auckland, New Zealand; ^6^Sports Performance Research Institute New Zealand, AUT Millennium, Auckland University of Technology, Auckland, New Zealand; ^7^Division of Training and Movement Sciences, University of Potsdam, Potsdam, Germany

**Keywords:** football, blood sample, monitoring, global positioning system, elite athletes

## Abstract

**Introduction:**

There is controversy in the literature in regards of the link between training load and injury rate. Thus, the aims of this non-interventional study were to evaluate relationships between pre-season training load with biochemical markers, injury incidence and performance during the first month of the competitive period in professional soccer players.

**Materials and Methods:**

Healthy professional soccer players were enrolled in this study over two pre-season periods. Data sets were available from 26 players during the first season (2014–2015) and 24 players during the second season (2015–2016) who completed two pre-season periods (6 weeks each). External training load was assessed from all athletes during training using Global Positioning System (GPS). Internal training load was monitored after each training session using rate of perceived exertion (RPE). Before and after each pre-season, blood samples were taken to determine plasma lactate dehydrogenase (LDH), creatine kinase (CK) and C-reactive protein (CRP). Injury incidence and overall performance (ranking of the team after the first five official games of the championship) were recorded for both seasons separately.

**Results:**

There was no statistically significant difference in mean RPE values of the two-preparation periods (2737 ± 452 and 2629 ± 786 AU, *p* = 0.492). The correlational analysis did not reveal significant associations between internal and external training load (RPE and GPS data) and biological markers. There was a significant positive correlation between RPE and LDH during the 2015/2016 season (*r* = 0.974, *p* = 0.001). In addition, a significant negative correlation was found between total distance >20 km/h and CRP during the 2015–2016 season (*r* = -0.863, *p* = 0.027). The injury rates for the two seasons were 1.76 and 1.06 per 1000 h exposure for the 2014–2015 and 2015–2016 seasons, respectively (*p* = 0.127).

**Conclusion:**

Our study showed that pre-season training load is not associated with overall team performance. This association is most likely multifactorial and other factors (e.g., technical and tactical level of the team, opponents, environment) may play an important role for the collective team performance. Our findings may help coaches to better prepare their athletes during pre-season.

## Introduction

Physiological demands in soccer have changed over the past years. In fact, the intensity level of soccer matches has increased tremendously (Carling et al., 2010; Clemente et al., 2013). Today, the average percentage of the maximum heart rate in football training varies between 63.5% (Clemente and Nikolaidis, 2016) and 87.1% (Suarez Arrones et al., 2014) depending on the expertise level. To withstand the demands of training and competition, high physical fitness levels are needed to cope with the increasing number of matches during a season and to prevent injuries. Nédélec et al. (2012) postulated that players from the Spanish national squad played on average 70 games during the 2009–2010 in preparation of the 2010 World Cup. Recently, studies used new technologies [e.g., Global Positioning System (GPS)] to analyze activity of soccer players during training and competition and to deduce information on underlying physical demands using these data sets (Carling et al., 2005; Carling, 2010; Nikolaidis et al., 2018). For instance, Bradley et al. (2013) reported that players in the English Premier League covered on average 681 m at running speeds ranging from 19.8 to 25.1 km/h. In addition, sprint distances of 248 m at velocities >25.1 km/h were registered during matches.

The number of the matches played, the intensity and the physical demands of these matches and related soccer training, may have an impact on fatigue (Clemente et al., 2017a,b) and soccer-related injuries. In this regard, Wong and Hong (2005), defined injuries in soccer players as “any condition that causes a player to be removed from a game, miss a game, or to be disabled enough to come to the medical tent.” The injury rates during competition ranged from 8.7 to 65.9 injuries per 1,000 h of exposure (Pfirrmann et al., 2016). In addition, Ekstrand (2016) analyzed the injury incidence rates among teams that played the Champions League during the 2015–2016 season and reported 2.3 injuries per 1,000 h of training and 20 injuries per 1,000 h of match exposure. Previously, several studies tried to examine the physiological causes of injuries in soccer. From an analysis of 6,000 injuries within 91 professional English football clubs (Hawkins et al., 2001), the majority of injuries were classified as strains (37%), as sprains (19%) and the lower extremity being the site of 87% of the injuries reported. Junge and Dvorak (2004), consider that 20–25% of all non-contact injuries are re-injuries of the same type and location. Negative adaptations to exercise training are dose related, with the highest incidence of illness and injuries occurring when training loads reach high levels (Foster, 1998; Owen et al., 2015). More recently, there is evidence that the ratio between acute and chronic loads is a better predictor for injuries compared with absolute training load (Gabbett and Whiteley, 2017; Jones et al., 2017). It has been proposed that the pre-competitive period is the period during a season with the highest training load. This was substantiated by Jeong et al. (2011), who showed that average physiological demands during the pre-season (mean heart rate 124 ± 7 beats/min; training load 4,343 ± 329 AU) were higher compared with in-season values (heart rate 112 ± 7 beats/min; training load 1,703 ± 173 AU). From this it follows that training load is higher and/or fitness level lower during the pre-season (Hawkins et al., 2001; Jeong et al., 2011). Several authors highlighted the impact of the pre-season training load on the incidence of soccer injuries. Hawkins et al. (2001), reported a greater proportion of overuse injuries, including tendinitis and paratendinitis, during the pre-season period compared with the in season (10.2 vs. 5.8%, *p* < 0.01) highlighting the need to further address this matter. Woods et al. (2002) were able to show that 17% of the overall number of injuries over the course of two seasons occurred during the pre-season. In addition, Noya Salces et al. (2014), observed that injury incidence during training was higher in pre-season and tended to decrease throughout the season (*p* < 0.05) in Spanish professional soccer players. More recently, Eliakim et al. (2018) in prospective study conducted within Israeli professional soccer team, demonstrated that an inappropriate pre-season training period as indicated by lower improvement in aerobic fitness was associated with a higher incidence of players’ injuries throughout the competitive soccer season. This suggests a possible role for high quality pre-season soccer training not only for shaping physical fitness, but also (among other factors) for injury prevention. However, the pre-season is considered to be the period with high training load and concomitant increased risk of sustaining injuries (Jones et al., 2017).

Besides the impact of training load on injuries, there is evidence that training load is also associated with physical stress during the preparation period (Clemente et al., 2017a). This has been established in soccer players through the assessment of blood markers as indicators for inflammation and muscle damage (Malone et al., 2018; Pascoal et al., 2018). Lactate dehydrogenase (LDH), creatine kinase (CK) and C-reactive protein (CRP) are biochemical markers often used to quantify muscle damage and inflammation (Djaoui et al., 2017). Anđelković et al. (2015) monitored these three blood parameters (CK, CRP, and LDH) during a competitive half season in elite soccer players. For instance, Pascoal et al. (2018) showed a 64% increase in CK over an 11-week preparation period in soccer players. However, to the best of the authors’ knowledge, no study compared two preparation periods monitored in the same soccer club and examined the influence of training load on injuries and overall performance during the first month of the first competitive period.

Therefore, the aims of this non-interventional study were to evaluate the effects of pre-season training load on biochemical markers, injuries and performance during the first month of the competitive period in professional soccer players. Hence, the possible link between the training load during pre-season period and collective performance during this period will be explored.

## Materials and Methods

### Participants

Initially, 35 professional soccer players were enrolled in this study. Full data sets were obtained from 26 players (age: 26.2 ± 5.1 years; height: 179.7 ± 5.1 cm; mass: 76.2 ± 5.7 kg) during the first season (2014–2015) and 24 players (age: 25.9 ± 5.2 years; height: 179.0 ± 5.6 cm; mass: 76.4 ± 5.6 kg) during the second season (2015–2016). A total of 14 players participated in both seasons. Coaches, strength and conditioning professionals, and the medical staff were the same during both seasons.

All participants were players from a second division French soccer club. Over the course of the study, players received a balanced training program with endurance, speed, agility, strength, technical, and tactical aspects that was delivered by a professional coach. All players were notified of the research protocol, benefits and risks before providing written informed consent. The protocol was fully approved by the Medical Center of Stade Lavallois Mayenne Football Club and ethics committee of the University of Rennes 2.

### Procedures

To study the training load during soccer, a non-interventional study was designed (i.e., no intervention during training) and data was collected during the pre-season of two competitive seasons (2014–2015 and 2015–2016). During the 5-week off-season, all players were asked to perform three sessions per week of unsupervised low volume and low intensity aerobic training. This is the traditional duration (4–6 weeks) of an off-season period in professional soccer (Silva et al., 2016). Two time points T1 (representing baseline conditions in June) and T2 (representing the end of the pre-training period in September) were considered as temporal benchmarks relevant to the soccer season. Blood samples were collected at each time point to monitor muscle damage (LDH and CK) and inflammation (CRP). Internal [ratings of perceived exertion (RPE)] and external training loads (GPS) were recorded after each training session and match during the two pre-season periods.

### Training Program

During pre-season, players performed on average between 6 and 8 training sessions and one game per week. This resulted in an overall weekly training and match exposure of 11 h. During in-season, 5–6 training sessions and one match per week were scheduled which amounted to an overall exposure of 8 h per week. In-season training sessions consisted of 30 min lower limb eccentric strength training and balance training followed by 60 min of soccer-specific technical and tactical drills including small-sided games, high intensity running and tactical exercises. Thus, training intensity and volume were higher during pre- compared with in-season. Training volume and intensity as well as recovery periods were individualized and could fluctuate from one session to the other. However, total training time was the same across the two seasons.

### Quantification of Training Load

#### Internal Training Load [Rating of Perceived Exertion (RPE)]

The training load was quantified on a daily basis by means of the session rating of perceived exertion (session-RPE) using Borg’s 6–20 scale (Foster, 1998; Foster et al., 2001). To ensure that the perceived exertion rating was reflective of the entire session rather than the last effort, data was collected 15–20 min following each training session. Prior to the start of the study, all players were familiarized with the Borg scale. Moreover, the included fitness coach and exercise scientist verified each player’s answers. The perception of effort was calculated according to Foster et al. (2001) in arbitrary units (AU). Rating of perceived exertion on the Borg scale was multiplied with effective duration (min) of a single training session.

#### External Training Load (Training and Match Exposure) [Global Positioning System (GPS)]

Data of training and match exposure were collected on a weekly basis. For team match exposure, the total match exposure time in hours for a team was calculated using the following formula:

$$({\mathrm{NM}}^{*}{\mathrm{PM}}^{*}\mathrm{DM})/60.$$

Where NM is the number of team matches played per week, PM is the number of players on the team (normally 11) and DM represents match duration in minutes (normally 90) (Fuller et al., 2006).

For team training exposure, the total training exposure time in hours was computed using the sum of the values for (PT^∗^DT)/60 for every training session throughout the study. PT is the number of players attending a training session and DT is the duration of a single training session in minutes (Fuller et al., 2006).

Total distance covered and distances covered at different intensities were collected on a daily basis using GPS technology (GPSPORT, 15 Hz). The selected running speed intensities were <12 km/h; 12–16 km/h; 16–20 km/h; 20–25 km/h and >25 km (Bradley et al., 2013). The transmitters were installed on the players just before each session and removed immediately afterward. GPS data were analyzed immediately after each session.

#### Biological Analyses

Two blood samples were collected during each pre-season. The first one was considered as baseline. The second one was taken at the end of the pre-season period. Plasma CK, CRP, and LDH were measured. The test conditions were standardized. Training loads before the test days were kept low and the same procedures were applied before all test days. The blood samples were collected (in tubes containing EDTA) in the morning after an overnight fast and on the same day of the week (Wednesday at 8.00 am).

The blood samples were collected in tubes containing EDTA and centrifuged for 10 min at 4° and 3000 rpm and the plasma was stored frozen at -80°C until the final analysis. The CRP, CK, and LDH activities were determined using a multiparametric analyzer (Konelab 30^TM^, Thermo Electron Corporation). CRP activity was determined using the immunoturbidimetry method. The intra-assay coefficient of variation for the CRP kit was 1.7%. CK activity was determined by the UV method (IFCC) using the N-acetyl-cysteine. The intra-assay coefficient of variation for the CK kit was 1.8%. LDH activity was determined by applying the enzymatic rate method (IFCC). The intra-assay coefficient of variation for the LDH kit was 1.1%.

#### Assessment of Injury Rates Over the Two Pre-season Periods

The medical staff of the soccer team reported and validated each injury in accordance with the Fédération Internationale de Football Association (FIFA) Consensus Statement (FIFA Consensus, 2006). The protocol was used to record the type, location, and severity of each injury. Responsible researchers and medical staff checked the database on a weekly basis.

In addition, exposition time was individually registered as each player’s participation during training and matches. The FIFA standard injury form used to record players injuries was received on a weekly basis from the medical staff team. Recorded injuries included any event resulting in the player being unable to train fully or to play matches (time-loss injuries) and the player was considered injured until the team’s medical staff allowed return to training and competition.

#### Location of Injuries

In this study, we used the following 12 categories to document location of injuries. Foot, ankle, lower leg, knee, thigh, hip/groin, upper extremities shoulder/clavicle, lumbar/sacrum/pelvis, head/face/neck/cervical, abdomen and sternum/rib/dorsal. This procedure has been applied in previous studies (Hawkins et al., 2001; Woods et al., 2002; Fuller et al., 2006).

#### Types of Injury

Injuries were classified into seven categories in accordance with the Consensus Statement for soccer (Fuller et al., 2006). These included fractures and bone stress joints (non-bone) and ligaments, muscles and tendons, contusions, lacerations and skin lesions, central/peripheral nervous system and other injuries.

In addition, injuries were also classified as traumatic (those with an acute onset) or overuse injuries (those without any known trauma).

The severity of each injury was defined according to the number of days elapsed from the date of injury to the date of the player’s return to full participation in team training or availability for competition. The injury severity was classified into four categories that have been used in previous studies (Fuller et al., 2006; Clarsen et al., 2012): minimal (≤3 days). mild (4–7 days), moderate (8–28 days) and severe (>28 days). In addition, recurrent injuries defined as injuries of the same type and location that occurred after the player recovered and returned to full participation were recorded. Recurrent injuries were classified as less severe equally severe or more severe in comparison to the original injury (Clarsen et al., 2012).

Injury rate was calculated as the number of injuries per 1000 h of exposure (Fuller et al., 2006).

#### Overall Performance

To quantify overall performance, results of the team during the first five games of the competitive period were considered (ranking. point’s won. goals scored. goals conceded). The first five games of the season are indicative of the overall performance of the competitive start of the season and it may therefore reflect the training effect of the pre-season period. Kelly and Coutts (2007) showed that three factors primarily determine intensity level of a match. These are (a) performance level of the opponent, (b) the number of training days during the week, and (c) match location. With regards to the level of the opponent, we cannot ascertain that the performance level was similar between seasons. However, all teams played in the same professional league. The number of days between games was the same for all teams at the beginning of the two seasons. During the first season, the team played three times at home and twice away. During the second season, the team played twice at home and three times away.

### Statistical Analyses

Results are expressed as means and standard deviations (SD). The statistical analyses were performed using Statistical Package for Social Sciences for Windows version 16.0 (SPSS Inc. Chicago). The power analysis (point biserial model) was computed with an assumed Type I error of 0.05, a Type II error rate of 0.20 (80% statistical power), and a large effect size based on a previous study with similar study design from Thorpe and Sunderland (2012) who observed a significant and large sized correlation between the number of sprints performed during a match and CK values (*r* = 0.88, *p* = 0.019). The analysis revealed that 26 participants would be sufficient to conduct our study. All included variables were tested for normality of distribution before analysis using the Kolmogorov-Smirnov test. Student’s *t*-test for independent samples was applied to contrast all variables between the two pre-season periods. All participants were included in a two-way analysis of covariance (ANCOVA) to estimate training load effects on the respective outcome variables (LDH, CK, and CRP). Baseline values were used as covariates. Injury rates were calculated as the number of injuries per 1000 h of training and match exposure (Fuller et al., 2006). Significant differences were assumed when *p* < 0.05. Effect sizes were calculated by converting partial eta-squared to Cohen’s *d* to document the size of the statistical effects observed and defined as small (0.00 ≤*d* ≤ 0.49), medium (0.50 ≤*d* ≤ 0.79), and large (*d* ≥ 0.80). Correlations between the independent variables LDH, CK, and CRP and the dependent variables external training load (i.e., GPS data) and internal training load (i.e., RPE) were determined using simple regression. The magnitude of the effect for the correlations was determined using the modified scale as proposed by Hopkins: *r* < 0.1, trivial; 0.1–0.3, small; >0.3–0.5, moderate; >0.5–0.7, large; >0.7–0.9, very large; >0.9, nearly perfect; and 1 perfect (Hopkins, 2002).

## Results

### Overall Performance

In terms of overall performance, statistically significant differences were found between the two seasons (Table 1). After the first five soccer matches of the season, there was a difference of 6 points and 12 places in the table in favor of the second season (*p* = 0.022, *d* = 0.332, small). More, while no win was recorded during the first five games of the first season, three of the first five games were won in the second season. Finally, the average goal was positive during the 2015–2016 seasons which was not the case during the 2014–2015 season.

**Table 1:** Statistics of the soccer team after the first 5 matches during the two seasons 2014–2015 (*n* = 26 players) and 2015–2016 (*n* = 24 players).

Season	Rank /20 teams	Pts	Matches	Win	Draw	Lost	Goal +	Goal -	Difference
2014–2015	16	4	5	0	4	1	2	3	–1
2015–2016	4	10	5	3	1	1	7	5	2

### RPE and GPS Data

There were no statistically significant differences between the two seasons in regards of external training load (GPS data, Table 2) and internal training load (2737 ± 452 and 2629 ± 786 AU; *p* = 0.492, *d* = 0.109, small). The daily mean training internal load (RPE) of the team was 456 for the 2014–2015 and 438 AU for the 2015–2016 preseason (*p* = 0.465, *d* = 0.235, small). The highest internal training loads were achieved during the second week of both pre-seasons (4060 AU for the first season (*p* = 0.006, *d* = 0.577, medium) and 4789 AU for the second season (*p* = 0.005, *d* = 0.601, medium). During the 2014–2015 season, total distance covered at running velocities >12 km/h was highest in week 3 (*p* = 0.032, *d* = 0.453, small). During the 2015–2016 season, the highest value was reached in week 4 (*p* = 0.039, *d* = 0.399, small) (2527 m and 2432 m).

**Table 2:** Indicators of external (GPS data) and internal load (RPE) of players determined through the 6 weeks of the pre-season periods in 2014–2015 (*n* = 26 players) and 2015–2016 (*n* = 24 players).

Indicators of external load (GPS data)				
	12–16 km/h	16–20 km/h	>20 km/h	TD > 12 km/h (m)	Indicators of internal load (Session RPE)
**Week 1**
2014–2015	965.2 ± 189.2	162.8 ± 44.8	46.7 ± 18.4	1174 ± 252.6	2315 ± 424.4
2015–2016	581.7 ± 115.3	132.7 ± 23.1	9.3 ± 4.3	723.6 ± 142.7	1657 ± 474.5
**Week 2**
2014–2015	1812.0 ± 727.7	483.0 ± 240.7	207.5 ± 90.3	2503 ± 1058.8	4060 ± 688.4
2015–2016	1325 ± 309	455.3 ± 66.5	214.1 ± 61.1	1994.3 ± 436.2	4789 ± 1263
**Week 3**
2014–2015	1528 ± 218.4	682.4 ± 126.3	316.2 ± 37.2	2527 ± 382	2772 ± 405.2
2015–2016	1180.6 ± 394	435.7 ± 145.2	192.2 ± 64.1	1808.5 ± 602.8	2828 ± 546.7
**Week 4**
2014–2015	851.0 ± 279	410.5 ± 248.4	299.3 ± 230.6	1561 ± 759	2462 ± 394.7
2015–2016	1131.6 ± 377	477 ± 159	239 ± 79.6	1847.5 ± 615.8	2003 ± 443.4
**Week 5**
2014–2015	1069.9 ± 142.9	340.8 ± 54.3	208.8 ± 75.2	1619 ± 272.4	2579 ± 236.1
2015–2016	934 ± 311.3	343.8 ± 114.6	234.5 ± 78.2	1512.2 ± 504.1	2583 ± 645.8
**Week ± 6**
2014–2015	676.1 ± 237.4	250.9 ± 58.7	155.6 ± 50.84	1082.6 ± 346.9	2233 ± 363
2015–2016	935.7 ± 311.9	319.3 ± 106.4	159.9 ± 53.3	1415 ± 471.7	1914 ± 266.4
**Means**
2014–2015	1150.4 ± 432.6	388.4 ± 183.3	205 ± 98.8	1744.4 ± 632.6	2737 ± 452
2015–2016	1014 ± 259.8	360.6 ± 128.1	174.9 ± 86.2	1550.2 ± 459.4	2629 ± 786
*P*-value	0.345	0.788	0.698	0.954	0.492

*TD, total distance; RPE, ratings of perceived exertion; AU, arbitrary unit.*

### Biological Data

There were no significant differences in CK and CRP development from the beginning to the end of the each pre-season (*p* > 0.05) (Table 3). However. LDH significantly increased from 174.37 ± 25.04 to 212.55 ± 34.81 UI.L^-1^ (*p* = 0.007, *d* = 0.904, large) during the 2015/2016- preparation period.

**Table 3:** Biological concentrations (LDH, CK, and CRP) of soccer players determined before and after the pre-season periods 2014–2015 and 2015–2016.

	2014/2015		*P*-value	Effect size	2015/2016		*P*-value	Effect size
	(*n* = 26 players)				(*n* = 24 players)			
	Before	After			Before	After		
LDH (UI.L^-1^)	188.9 ± 30.0	190.1 ± 32.4	0.791	0.874	174.4 ± 25.0	212.6 ± 34.8	0.007**	0.904
CK (UI.L^-1^)	339.9 ± 178.0	413.9 ± 335.6	0.290	0.393	256.1 ± 170.7	380.2 ± 176.1	0.079	0.433
CRP (mg.L^-1^)	0.4 ± 0.4	0.79 ± 1.08	0.130	0.207	1.2 ± 1.9	0.9 ± 0.5	0.569	0.197

*Data are presented as mean ± SD. ^∗∗^Significant difference between before and after season period with *p* < 0.01.*

### Correlations Between the Parameters Measured

Eight injuries were recorded during both periods (5 in 2014–2015 and 3 in 2015/2016). The injury rates for the two seasons amounted to 1.76 and 1.06 for 2014/2015 and 2015/2016, respectively (Table 4). The difference between the two seasons was not statistically significant (*p* = 0.127, *d* = 0.339, small). Table 5 contains type and duration of injuries recorded during the two seasons.

**Table 4:** Exposure time during training and matches and injury rates during the two pre-season periods 2014–2015 (*n* = 26 players) and 2015–2016 (*n* = 24 players) and during the first five matches of the season.

Pre-season periods	Number of injuries	Exposure time training^(a)^	Exposure time match^(b)^	Injury rate^(c)^
2014–2015	5	2664	165 165	1.76
2015–2016	3	2664		1.06

*^*(a)*^Exposure training time calculated using number of players per session (around 24 players). Number of training session (around ± 74 for the studied period. 12 weeks) duration of session per min (90 min). ^*(b)*^Exposure match time was calculated using of number of played matches (10 matches for the examined period). Number of players during the game (11 players) and duration of match per min (90 min). ^*(c)*^Injury rate was calculated as the number of injuries divided by hours of exposure and multiplied with 1000.*

**Table 5:** Type and duration of injuries recorded during the two pre-season periods and the five first matches of the season.

	2014–2015		2015–2016	
Traumatic injuries	3		0	
Non-traumatic injuries	2		3	
Total	5		3	

	**Traumatic**	**Non-traumatic**	**Traumatic**	**Non-traumatic**

Average duration (days)	23.0 ± 31.2	27.5 ± 14.9	0	52.3 ± 54.0
	All injuries		All injuries	
	24.8 ± 23.4		31.9 ± 36.4	

	**Traumatic**	**Non-traumatic**	**Traumatic**	**Non-traumatic**

Total cumulative duration (days)	69	55	0	157
	All injuries		All injuries	
	124		157	

**FIGURE 1:**
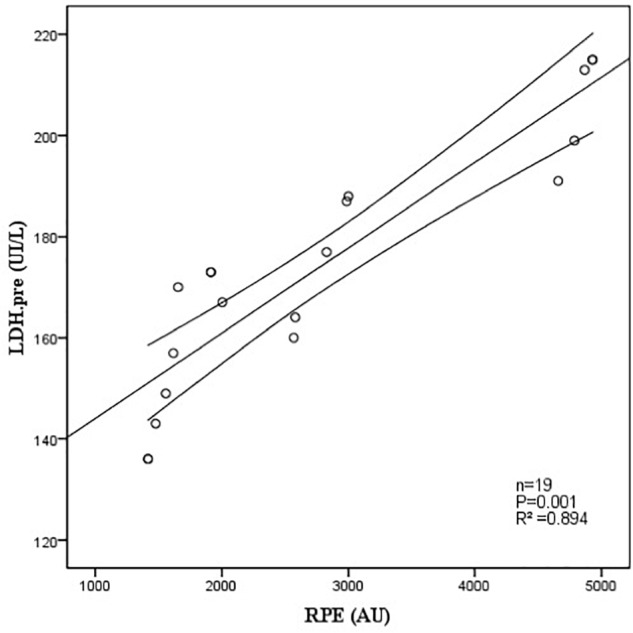
Relationship between RPE and LDH determined before the pre-season period 2015/2016 (*N* = 17; *r =* 0.974. *p* = 0.001, nearly perfect).

**FIGURE 2:**
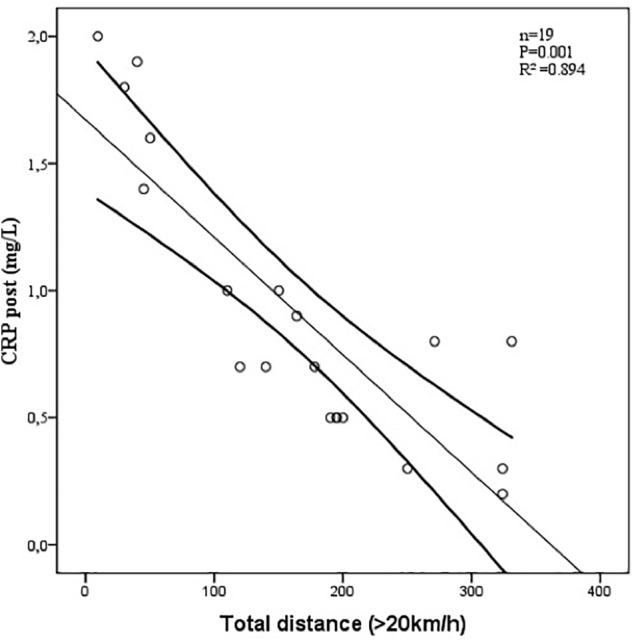
Relationship between total distance >20 km/h and CRP determined after the pre-season period 2015/2016 (*N* = 19; *r* = –0.863, *p* = 0.027, very large).

The relationship between parameters of external and internal training load and biological markers are presented in Table 6. Only two statistically significant correlations were found. In fact, there was a near perfect correlation between RPE and LDH PRE during the 2015/2016 season (*n* = 17; *r =* 0.974, *p* = 0.001, nearly perfect) (Figure 1). There was a significant negative correlation between total distance >20 km/h and CRP POST during the 2015/2016 season (*n* = 19; *r* = -0.863, *p* = 0.027, very large) (Figure 2).

**Table 6:** Relationship between external (GPS data), internal indicators (RPE) and biological parameters (LDH, CPK, and CRP).

Indicators			LDH PRE	LDH POST	CK PRE	CK POST	CRP PRE	CRP POST
Session RPE	2014/2015	*r*	0.736	0.757	0.041	–0.129	0.008	0.733
		*P*	0.095	0.082	0.939	0.807	0.989	0.097
	2015/2016	*r*	0.974**	0.510	0.179	0.353	0.394	–2.211
		*P*	0.001	0.734	0.734	0.492	0.440	0.688
Total distance >12 km/h	2014/2015	*r*	–0.205	0.043	–0.739	–0.680	–0.510	0.266
		*P*	0.696	0.935	0.093	0.137	0.310	0.611
	2015–2016	*r*	0.004	–0.468	–0.270	–0.359	–0.688	–0.658
		*P*	0.994	0.350	0.605	0.484	0.131	0.155
Total distance 12–16 km/h	2014/2015	*r*	0.056	0.425	–0.570	–0.583	–0.443	0.656
		*P*	0.916	0.400	0.238	0.224	0.379	0.157
	2015/2016	*r*	0.103	–0.302	–0.151	–0.299	–0.599	–0.559
		*P*	0.846	0.561	0.776	0.565	0.209	0.249
Total distance 16–20 km/h	2014/2015	*r*	–0.421	–0.280	–0.714	–0.596	–0.407	–0.147
		*P*	0.406	0.591	0.111	0.212	0.423	0.781
	2015/2016	*r*	–0.039	–0.511	–0.309	–0.381	–0.701	–0.633
		*P*	0.942	0.300	0.551	0.457	0.120	0.177
Total distance >20 km/h	2014/2015	*r*	–0.419	–0.565	–0.572	–0.452	–0.381	–0.434
		*P*	0.408	0.243	0.235	0.368	0.456	0.390
	2015/2016	*r*	–0.229	–0.808	–0.517	0.438	–0.803	–0.863*
		*P*	0.662	0.052	0.293	0.385	0.055	0.027

*r: correlations of Pearson; *p*: bilateral difference; ^∗^Significant correlations. ^∗∗^*p* < 0.01 and ^∗^*p* < 0.05.*

No significant correlation was observed between the training load and the overall performance of the team and the injury rates recorded during the two pre-seasons.

## Discussion

The aim of this study was to examine the effects of pre-season training load on biochemical markers, injury incidence and performance during the first month of the competitive period in professional soccer players. To our knowledge, this is the first attempt to investigate this issue in a professional soccer team. Our findings imply that training load during the pre-season does not have an effect on overall team performance during the first five official matches of the season. Moreover, no significant relationship was found between training load and injury incidence, inflammation and muscle damage markers. Thus, skill-related (technical) and tactical factors may play a role in the collective performance during the first month of the competitive season.

### Training Load

Internal training load was similar during the two pre-seasons (456 AU in 2014–2015 vs. 438 AU in 2015–2016). Other researchers found similar values in professional soccer players. For instance, Malone et al. (2015), analyzed internal training load (i.e., RPE) among professional English soccer players and observed that during the pre-season, the training load amounted to 447 ± 209 AU. There is equivocal data in the literature with some reporting similar results (Redkva et al., 2017), and others reporting lower values (321 AU for Jeong et al., 2011 and for Clemente et al., 2017a, 308 AU (1 match per week) and 245 AU (2 matches per week). However, these discrepancies in findings can most likely be explained by the fact that Jeong et al. (2011) used data from only 1 week during the pre-season. Clemente et al. (2017a) monitored the internal training load in professional soccer players from the Portuguese premier league during one season including the pre-season period.

Global Positioning System data from this study were similar to those reported by other authors. Scott et al. (2013) monitored external training load in professional soccer players and observed distances of 544 m per session at a high running velocity (>14 km/h) and 132 m per session at a high running velocity of >19.8 km/h. More recently, Clemente et al. (2018) monitored one typical training week during the in-season period in two professional teams from Portugal and the Netherlands. These authors measured average distances of 585 and 213 m, respectively for velocities of 14–20 km/h and >20 km/h. In our study, distances at these intensities were even higher compared with Scott et al. (2013) and lower compared with Clemente et al. (2018). Data amounted to 388 and 205 m for the 2014–2015 and 360 and 175 m for the 2015–2016 seasons, respectively. These differences can mainly be explained by several experimental factors such as the period of the monitored season and the applied test device (e.g., GPS).

### Overall Performance

In the current study, the first five games of the season were chosen to represent the overall performance. Team performance was largely different between the two seasons while training load was not (Table 1). Soccer match-performance seems to depend on the successful interaction of physical, tactical and technical characteristics of the game (Carling, 2010). In addition, the performance level of the opponent may also have an impact on match performance even though the teams were from the same league. Fatigue-related declines in physical performance have been reported during a soccer match. In fact, Carling et al. (2010) showed that the covered sprint distances at high intensity decreased from the first 15 min to the last 15 min of a match (total high intensity sprinting: 468 m vs. 411 m). Of note, technical performance during the match was not affected even if three matches were played within ± 7 days (Carling et al., 2010). In addition, Janković et al. (2011) showed that the covered distances during a match are not associated with the final results of the match. Technical parameters of the players and the tactical efficiency appear to be of larger importance for the outcome of a soccer match. Top teams score on average more goals relative to the number of shots on the goal (Lago, 2009). According to the same authors, better teams also have higher rate of ball possession. They showed that ball possession was an important parameter for the match result. Saito et al. (2013) showed that the number of passes and especially, the number of pass correctly carried out had an importance on the result of the match. Therefore, more than physical fitness, technical, tactical and efficacy parameters together with the performance level of the opposing team appear to have a major impact on the collective team performance that is characterized by the match results.

### Relationship Between Training Load and Injury Incidence

In this study, the average of reported training injuries amounted to 1.76 per 1,000 h exposure in the seasons 2014–2015 and 1.06 per 1,000 h of exposure in 2015–2016. These injury rates were recorded during July and August of each season and were below those that were recently reported for professional soccer players. In fact, Hawkins et al. (2001) showed a greater proportion of overuse injuries (e.g., tendonitis and paratendonitis) during the pre- compared with the in-season (10.2% vs. 5.8%, *p* < 0.01). Moreover, Woods et al. (2002) emphasized that over the course of two soccer seasons, 17% of the overall number of injuries occurred during the pre-season. In addition, Noya Salces et al. (2014) reported higher injury incidence rates during training in the pre-season compared with the rest of the season in Spanish professional soccer players. Ekstrand (2017), showed that during the 2016–2017 season, injury rates in professional European soccer leagues averaged 2.3 injuries per 1,000 h of training. However, in professional soccer players from Israel, Eliakim et al. (2018) reported higher match injury rates (9.4 per 1,000 match hours) compared with injuries sustained during training (4.7 per 1,000 training hours) (Eliakim et al., 2018). These authors further postulated that most of the recorded injuries were overuse injuries of the lower limbs (71%) (Eliakim et al., 2018).

The number of injuries recorded in the current study was relatively low and did not allow us to detect the role that players’ absence could have on collective performance. Training load on both pre-season periods seemed to have a little impact on injuries, since no significant relationship was observed. However, recently, Eliakim et al. (2018) examined the effect of pre-season fitness on injury rate during two consecutive competitive seasons among Israeli professional soccer players. These authors observed that although there were no differences in initial fitness characteristics (in the beginning of pre-season training) between injured and non-injured players, improvements in VO2 max during the pre-season training period were significantly lower among injured players compared to non-injured players.

In the current study, the injury rate over the two seasons was lower than two injuries per 1,000 h of exposure (Table 5). Further, we were not able to detect significant correlations between injury rates overall team performance. Thus, the rather low injury rate during the season 2015–2016 does not appear to play a major role in the collective performance during competition. Dauty and Collon (2011), also showed that there was no relationship between the number of injuries and the final ranking in a professional soccer French team lower injury incidence was strongly correlated with team ranking position (Eirale et al., 2013). Furthermore. Hägglund et al. (2013), reported a strong correlation between number of injuries and final ranking after having followed 24 professional soccer teams over 11 years.

### Biological Follow-Up

The results of inflammation and muscle damage markers measured in this study were similar to those of the data reported in the literature. These experiments were conducted during the pre-season (Djaoui et al., 2017). In our study, the increase of the different parameters was not significant unlike other studies. This divergence could mainly be explained by the fact that in the current study for technical reasons, the blood samplings were done 1 week after the end of the pre-season period, which may be sufficient to allow the various markers to recover their initial values. It is also possible that the little variability between the different training weeks does not allow for a large variation in blood markers. Hence, another reason may be the great heterogeneity of the team. However, in the current study, a significant and nearly perfect correlation was found between RPE and LDH before training started in the 2015/2016 season. In addition, a significant and very large association was observed between total distance covered (>20 km/h) and CRP after the pre-season period 2015/2016. Several studies showed that intensified training or match exposure could influence the increase of inflammation and muscle damage markers (Nédélec et al., 2012; Malone et al., 2018; Souglis et al., 2018). In this regard, the first training session after the off-season may induce microtrauma (muscle damage) to structural and contractile components within the muscle fiber. This again results in increased LDH and may affect players’ perceived exertion, even if training intensity was kept rather low during this early stage of the season (Meister et al., 2014). It is well known that running speeds, accelerations and decelerations over a certain magnitude (moderate to high) and over a certain period may induce muscle inflammation as indicated by the CRP values (Young et al., 2012).

### Limitations, Strengths and Practical Applications

Several limitations should be acknowledged in the current study: (i) blood samples were taken 1 week after the end of the training period and only three blood markers were measured. However, more markers should be monitored over the entire duration of the season (Djaoui et al., 2017). (ii) During the two examined seasons, only a single competitive soccer team was monitored. In addition, there was fluctuation in the number of the players over the two seasons due to traded players. Moreover, only the first five games of the season were computed to analyze overall team performance. It will be interesting to monitor several teams from different countries and championships and to extend the analysis to one or more entire seasons; and (iii) due to a relatively small cohort, the rather low overall number of injuries may have failed to highlight a relationship between training load and injuries. Monitoring injuries and training load during the entire season from more teams may provide useful results for strength and conditioning coaches in soccer and medical staff (Soligard et al., 2016; Foster et al., 2017).

The monitoring of the training load in professional soccer players using, for example, simultaneous measures of GPS and RPE, remains the best way to track internal and external training load. Training load during the pre-season appears to influence team results to a lesser extend than the technical and tactical level of the team. This study may suggest that the technical level and the quality of the players in a team are essential factors with regards to the collective performance.

## Conclusion

The results of this study showed that training load during the pre-season period was not related to overall performance and injury rates of professional soccer players during the first months of the competitive season. With reference to our findings, it can be hypothesized that technical and tactical factors together with performance level of the opponent could have an impact on success in competition. More research with larger cohorts is needed to verify these findings.

## Ethics Statement

The protocol was fully approved by the Medical Center of Stade Lavallois Mayenne Football Club and Ethics Committee of the University of Rennes 2.

## Author Contributions

SC, GR, and HZ conceived and designed the research. SC, GR, and HZ conducted the experiments. IS, SZ, AZ, and ABA analyzed the data. SC, AA, MB, UG, ABA, SZ, AZ, and HZ wrote the manuscript. All authors read and approved the manuscript.

## Conflict of Interest Statement

GR was employed by company Stade Lavallois Mayenne Football Club. The remaining authors declare that the research was conducted in the absence of any commercial or financial relationships that could be construed as a potential conflict of interest.
